# A comparison between heart rate and heart rate variability as indicators of cardiac health and fitness

**DOI:** 10.3389/fphys.2013.00337

**Published:** 2013-11-20

**Authors:** Catharina C. Grant, Carien Murray, Dina C. Janse van Rensburg, Lizelle Fletcher

**Affiliations:** ^1^Section Sports Medicine, University of PretoriaPretoria, South Africa; ^2^Department of Statistics, University of PretoriaPretoria, South Africa

**Keywords:** autonomic cardiac control, prognostic indicators, exercise ability

## Abstract

Quantification of cardiac autonomic activity and control via heart rate (HR) and heart rate variability (HRV) is known to provide prognostic information in clinical populations. Issues with regard to standardization and interpretation of HRV data make the use of the more easily accessible HR on its own as an indicator of autonomic cardiac control very appealing. The aim of this study was to investigate the strength of associations between an important cardio vascular health metric such as VO_2_max and the following: HR, HRV indicators, and HR normalized HRV indicators. A cross sectional descriptive study was done including 145 healthy volunteers aged between 18 and 22 years. HRV was quantified by time domain, frequency domain and Poincaré plot analysis. Indirect VO_2_max was determined using the Multistage Coopers test. The Pearson correlation coefficient was calculated to quantify the strength of the associations. Both simple linear and multiple stepwise regressions were performed to be able to discriminate between the role of the individual indicators as well as their combined association with VO_2_max. Only HR, RR interval, and pNN50 showed significant (*p* < 0.01, *p* < 0.01, and *p* = 0.03) correlations with VO_2_max. Stepwise multiple regression indicated that, when combining all HRV indicators the most important predictor of cardio vascular fitness as represented by VO_2_max, is HR. HR explains 17% of the variation, while the inclusion of HF (high frequency HRV indicator) added only an additional 3.1% to the coefficient of determination. Results also showed when testing the normalized indicators, HR explained of the largest percentage of the changes in VO_2_max (16.5%). Thus, HR on its own is the most important predictor of changes in an important cardiac health metric such as VO_2_max. These results may indicate that during investigation of exercise ability (VO_2_max) phenomena, quantification of HRV may not add significant value.

## Introduction

It is known that cardiovascular disease (CVD) has reached pandemic proportions worldwide. It is identified as one of the most important causes of death in several countries including USA, Pacific and Middle Eastern countries, China and Europe (Barakat et al., 2012; Go et al., 2013). Factors, contributing to this pandemic is: smoking, physical activity/fitness, blood pressure, blood glucose, total cholesterol levels, weight, and diet. Of these factors, also called cardio vascular health metrics (Yang et al., 2012), the level of activity and fitness is a relative easy way to modify a person's CVD risk factor. However, low physical activity levels is also one of the most common problems identified across different age group contributing toward the CVD pandemic (Barakat et al., 2012). It is known that an increase in fitness/exercise capacity measured by VO_2_max, results in a reduction in CVD risk (Barakat et al., 2012). Active individuals present half the coronary artery disease risk compared to a very inactive group. Exercise and increased fitness not only help to prevent disease risk factors but can also treat existing risk factors such as high insulin levels, hypertension, hyperlipidaemia and obesity.

The link between cardiac autonomic neuropathy (increased sympathetic, decreased parasympathetic activity or a combination of the two), and CVD is well established (Lahiri et al., 2008). Quantification of cardiac autonomic activity and control via heart rate (HR) and heart rate variability (HRV) is known to provide prognostic information in clinical populations. HR is used as a CV mortality risk factor indicator and HRV as a predictor of disease outcome (Kannel et al., 1987; Bravi et al., 2011; Sacha et al., 2013).

Quantification of HRV, integrating the sympathetic and parasympathetic cardiac influences, is a complex measurement of the autonomic nervous system and its responses to internal and external stimuli (Lahiri et al., 2008). Issues with regards to standardization and interpretation (Grant et al., 2011) of HRV data make the use of the more easily accessible HR on its own as an indicator of autonomic cardiac control very appealing. It is important to establish if the more complex concept of HRV determination adds prognostic value above HR measurements. In an effort to quantify the contribution of HR to the prognostic value of HRV, the aim of this study was to investigate the strength of associations between an important cardio vascular health metric such as VO_2_max and HR, as well as between VO_2_max and HRV indicators including RMSSD, pNN50, LF, HF, LF/HF, SD1, and SD2 (abbreviations explained in Table 1). HR normalized HRV indicators were also calculated and used as regressors to determine if HR independent variability shows similar associations with VO_2_max than standard HRV indicators. Regression analyses were employed to determine the relative importance of HR and HRV indicators as predictors of an important cardio vascular health metric such as VO_2_max.

**Table 1:** **HRV indicators defined**.

**HRV indicator**	**Definition**
Mean RR (s)	The mean of the intervals between successive QRS complexes
RMSSD (ms)	Root mean square of the standard deviation between RR intervals
pNN50 (%)	The percentage of successive RR interval differences over the entire measurement larger than 50ms
LF Power (ms^2^)	Peak between 0.04 and 0.15Hz
HF Power (ms^2^)	Peak between 0.15 and 0.5Hz
LF/HF	LF Power (ms^2^) divided by HF Power (ms^2^)
SD1 (ms)	Indicator of the standard deviation of the instantaneous RR variability
SD2 (ms)	Indicator of the standard deviation of the continuous or long term variability of the heart rate

The primary hypothesis was that HR and RR intervals show stronger correlations with VO_2_max than the HRV indicators, and are on their own stronger predictors of VO_2_max than the more complex indicators of HRV (RMSSD, pNN50, LF, HF, LF/HF, SD1, and SD2). A secondary hypothesis was that, when including HR and HR normalized indicators of variability (RMSSD/RR, LF/RR^2^, HF/RR^2^, LF/HF, SD1/RR, SD2/RR) regression analysis will show that HR is still the most important predictor of VO_2_max.

## Materials and methods

### Background and determination of the health metric VO_2_max

The gold standard in fitness testing is generally regarded as the VO_2_max test for measuring aerobic fitness or cardiorespiratory endurance. VO_2_max is defined as the maximum volume of oxygen that can be utilized in 1 min during maximal exercise. It is measured in millilitres of oxygen used in 1 min per kilogram of body weight, (ml/kg/min) (Wilmore and Costill, 2004). Elite endurance athletes are known to have high VO_2_max values and it is thought that the more oxygen the body can use during strenuous exercise, the more energy it can produce (Wilmore and Costill, 2004).

VO_2_max can be measured directly or indirectly. The direct measurement needs to be done in a laboratory and is expensive, time consuming and requires special expertise. The test subject must reach his/her maximum work capacity in order to determine VO2 max accurately. The test is done on a treadmill or a bicycle and a strict protocol is followed. The athlete is required to exercise while the speed and intensity is gradually increased. The volume and oxygen concentration of the inhaled and exhaled air of the athlete is measured to determine how much oxygen is used. The oxygen consumption usually rises in direct relationship to the increase in exercise intensity up to a certain point. At this point the oxygen consumption reaches a plateau and does not rise further even with a further rise in intensity. This plateau marks the VO_2_max.

VO_2_max can also be determined indirectly, as an estimate of the true VO_2_max. The Cooper 12 min run test was used in the current study. This assessment uses a set period of time (12 min) and scoring according to distance (in meters). The participants were asked to run or walk for 12 min as fast as possible (Cooper, 1968). This test was performed on a 400 m tartan athletics track. A warm-up of light aerobic activities and flexibility exercises was performed for 5 min before the start of the test. Testers were placed at 100 m intervals around the track as a source of verbal motivation. A prediction of VO_2max_ from the distance covered at the end of the 12 min period was obtained by applying the distance run to the Cooper regression equation: VO_2_max (ml/kg/min) = 0.0268 (distance covered in meters)—11.3 (Cooper, 1968).

The sedentary person has a much lower VO_2_max generally compared to the active fit individual, with the elite endurance athlete having the highest VO_2_max. Genetics play a role in an individual's exercise ability but VO_2_max can be improved with physical training. The more unfit, the more the VO_2_max can be improved. As exercise capacity improves, skeletal muscle strength and endurance also improve (Paterson et al., 2004; Stringer, 2010).

### Study description

A hypotheses driven, cross sectional, descriptive study was performed. The study protocol was submitted and approved by the Ethics Committee of the University. A total of 145 healthy participants from a group of 235 volunteers were accepted to take part in the research study. Exclusion criteria included refusal to give voluntary written informed consent; a history of cardiovascular, hepatic, respiratory, or renal impairment, as well as pulmonary, metabolic, and orthopaedic diseases requiring medical attention; lung/respiratory tract infection in the previous 2 weeks; and medication that could influence cardiovascular control and psychological disorders. None of the participants were professional athletes or high-level sport participants. All participants gave written informed consent before commencement of the intervention. Participants fasted overnight and were asked not to use any caffeine, alcohol or to smoke 24 h prior to the measurements. Measurements were taken in a temperature regulated, quiet environment, in the morning before 12H00. The Polar 810E HR monitor system was used to record supine RR intervals for a 10 min period. The 5 to 10 min period of this recording was used to quantify the HRV. Computer software from the University of Kuopio, Finland calculated the HRV indicator values by time domain analysis (RR, STDRR, RMSSD, and pNN50), frequency domain analysis (LF, HF, and LF/HF), and Poincaré plot analysis (SD1 and SD2) (Mourot et al., 2004). HRV indicators determined are listed in Table 1; Mourot et al., 2004).

### Statistical analyses

The Pearson's correlation coefficient (*r*) was calculated. Correlation strength were defined as very low if rho is smaller than 0.2; low to moderate if rho is larger than 0.2 but smaller than 0.4; and moderate if rho is larger than 0.4 but less than 0.6 (Landis and Koch, 1977). The level of significance was set at the conventional 5%, thus, when *p*-values were less than 0.05, the associations were identified as statistically significant.

To test the hypotheses, linear regression was used to determine the most important predictors of changes in cardio vascular fitness and exercise ability as represented by VO_2_max. Indicators included were: HR, RR interval, RMSSD, pNN50, LF, HF, LF/HF, SD1, SD2. The following HR normalized HRV indicators were also included in a separate set of regression analyses: RMSSD/RR, LF/RR^2^, HF/RR^2^, LF/HF, SD1/RR, and SD2/RR. Both simple linear and multiple stepwise regressions were performed to be able to discriminate between the role of the individual indicators as well as their combined relationship with VO_2_max (Field, 1994).

## Results

A summary of the participants (*N* = 145) and HRV indicator values are displayed in Tables 2, 3.

**Table 2:** **Median, mean and standard deviation (*SD*) of participant body mass index (BMI) and exercise capacity (VO_2_max**).

	**Mean**	***SD***	**Median**
BMI (kg/m^2^)	23.07	2.36	22.62
VO_2_max (ml/kg/min)	49.54	8.79	53.53
2.4 km run time (s)	733.71	179.10	661.50

**Table 3:** **Mean, standard deviation (*SD*), median and inter quartile range of HRV indicators and normalized HRV indicators**.

	**Mean**	***SD***	**Median**	**Inter quartile range(Q1;Q3)**
Mean RR (ms)	894.10	137.03	884.50	186.25 (791.00;977.25)
Mean HR (beats per minute)	69.22	10.5	68.78	14.61 (61.83;76.44)
RMSSD (ms)	76.04	44.84	65.00	53.25 (43.40;96.65)
pNN50 (%)	39.08	22.84	38.30	39.85 (18.40;59.25)
LF (ms^2^)	1148.19	1291.74	999.00	1336 (582.50;1918.50)
HF (ms^2^)	2760.15	3465.31	1721.00	2627 (582.50;1918.50
LF/HF	0.81	0.58	0.67	0.74 (0.35;1.09)
SD1(ms)	54.06	31.83	46.10	37.80 (30.95;68.75)
SD2(ms)	90.48	37.17	83.10	51.60 (64.00;115.60)
**NORMALIZED HRV INDICATORS**				
RMSSD/RR	0.08	0.04	0.07	0.05 (0.05;0.10)
LF/RR^2^	0.0017	0.0013	0.0012	0.001 (0.001;0.002)
HF/RR^2^	0.0031	0.0034	0.0020	0.002 (0.001;0.003)
SD1/RR	0.060	0.030	0.050	0.04 (0.03;0.07)
SD2/RR	0.10	0.040	0.090	0.05 (0.07;0.12)

The HR, RR intervals, and HRV indicators (normalized and non-normalized) were compared to the VO_2_max of participants results, to establish any significant relationships (Table 4).

**Table 4:** **Pearson's correlation coefficients (*r*) and significance (*p*-value) for VO_2_ max and HRV indicators**.

**HRV Indicators**	**Indirect VO_2_max *r* (*p*-value)**	**Normalized HRV indicators**	**Indirect VO_2_max *r* (*p*-value)**
Mean RR	0.41(< 0.01)^*^	NA	NA
Mean HR	−0.41 < 0.01^*^	NA	NA
RMSSD	0.09 (0.29)	RMSSD/RR	0.02 (0.80)
pNN50	0.19 (0.03)^*^	NA	NA
LF	0.10 (0.21)	LF/RR^2^	0.02 (0.95)
HF	0.001 (0.99)	HF/RR^2^	−0.03 (0.72)
LF/HF	0.02 (0.82)	NA	NA
SD1	0.09 (0.29)	SD1/RR	0.02 (0.80)
SD2	0.14 (0.10)	SD2/RR	0.02 (0.79)

NA, Not applicable;

* *, significant: p < 0.05*.

The results shown in Table 4 indicate that only one HRV indicator value, pNN50, correlated significantly with VO_2_max. When indicators were HR normalized by dividing by RR or RR^2^, no significant correlations were found. All significant correlation coefficients were between 0.19 and 0.41 indicating very low to moderate associations.

Simple linear regression analyses (Table 5) showed that only HR, RR, and pNN50 explained a significant (*p* < 0.05) proportion of the variation in VO_2_max. Stepwise multiple regression (Table 5) indicated that, when including all nine indicators (HR, RR, RMSSD, pNN50, LF, HF, LF/HF, SD1, SD2), the statistical model containing a linear combination of HR and HF, explained the largest proportion of the variation, i.e., is the best predictor of variation in VO_2_max 20.1%). However, HR entered the model first and by itself explains 17% of the variation, while the inclusion of HF added only an additional 3.1% to the coefficient of determination. Stepwise multiple regression also showed that, when including HR and all normalized HRV indicators (RMSSD/RR, LF/RR^2^, HF/RR^2^, LF/HF, SD1/RR, SD2/RR), the statistical model consisting of a linear combination of HR and HF/RR^2^, will be the best predictor of variation in VO_2_max, that is 19.0%. HR on its own explained 16.5% and a combination of HR and HF/RR^2^ thus, explained only a further 2.5%.

**Table 5:** **Simple and stepwise multiple linear regression results**.

**Regression type**	**Predictors of VO_2_max included**	**Significant predictors (*p* < 0.05)**	**Coefficient of determination R^2^**
Simple Regression	HR	HR	0.170
Simple Regression	RR	RR	0.161
Simple Regression	pNN50	pNN50	0.035
Multiple Regression	Nine indicators HR, RR, RMSSD, pNN50, LF, HF, LF/HF, SD1, SD2	HR and HF	0.201
Multiple Regression	HR and six normalized HRV indicators: HR/RR, RMSSD/RR, LF/RR^2^, HF/RR^2^, LF/HF, SD1/RR, SD2/RR	HR and HF/RR^2^	0.190

## Discussion

The primary hypothesis was that straightforward measurements such as HR and RR intervals show stronger correlations with VO_2_max than the HRV indicators, and is on their own stronger predictors of VO_2_max than the more complex indicators of HRV. Study results partially confirmed the first hypotheses. Moderately strong correlations were found between HR and RR intervals vs. VO_2_max. The only other correlation found (pNN50) was of very low strength (*r* = 0.19). The stepwise multiple regression analysis indicated that HR predicted 17%, and the combination of HR and HF predicted 20.1% of the variation in VO_2_max, when entering the following HRV indicators: HR, RR, RMSSD, pNN50, LF, HF, LF/HF, SD1, SD2. With regards to the second hypothesis multiple *r*egression analysis showed that the combination of HR and HF/RR^2^ explained only 2.5% more of the variation in VO_2_max, than HR alone (16.5%), when including all normalized indicators.

Recent publications by Sacha et al. (2013) suggested that, because of physiological and mathematical relationships between HR and HRV, the dependence of HRV indicator values on HR can be modified by mathematical manipulation. This is due to the known fact that RR variation is significant higher for a slow average HR than for higher mean HR. Following suggestions by Sacha et al. (2013) to minimize the HRV indicator*'*s dependence on HR, correlational and regression statistical analyses in this study were performed firstly with the actual HR, RR, and HRV indicator values and again after each were normalized by dividing by the mean RR or RR^2^.

Correlations found between the HR, RR intervals, pNN50, and VO_2_max were significant (*p* < 0.05), but of very low to medium strength (0.19 ≤ *r* ≤ 0.41). A significant positive correlation was found between mean RR interval and VO_2_max (*p* < 0.01; *r* = 0.41) and as expected a significant negative correlation between mean HR and VO_2_max (*p* < 0.01; *r* = −0.41). These associations concur with some previous study findings. Higher fitness is associated with a lower HR, higher VO_2_max, and also higher vagal cardiac control (Nagai et al., 2004; Spierer et al., 2007; Gilder and Ramsbottom, 2008). The strength of the associations found in this study are lower than values reported in literature but this may be due to the fact that VO_2_max was only indirectly determined and not directly with gas analyses.

The fact that only one HRV indicator (pNN50) correlated significantly with VO_2_max, may indicate that correlations between HRV indicators and VO_2_max exist mainly due to the relationship between HR (independent of variability) and VO_2_max.

Regression analysis confirmed that, when including HR, RR, RMSSD, pNN50, LF, HF, LF/HF, SD1, and SD2, the most important predictor of cardio vascular fitness and exercise ability as represented by VO_2_max, is HR. Results also showed when testing the normalized indicators, HR explained the largest percentage of the changes in VO_2_max. Thus, HR on its own is the most important predictor of changes in an important cardiac health metric such as VO_2_max. Thus, the more complex concept of HRV quantification unlock only a small percentage of additional information with regards to autonomic function, compared to information obtained from only HR and RR interval measurements. These results may indicate that during investigation of exercise ability *(*VO_2_max) phenomena, quantification of HRV may not add significant more value. This is a novel idea which should be investigated further in clinical populations.

Limitations of this study include the fact that VO_2_max was not directly determined with gas analyses, but only indirectly determined. Another drawback is that the tachogram used to determine HRV, was sampled only in a resting, supine position. Previous studies indicated that HRV measured during a stressor, such as standing upright, show more significant correlations with cardiopulmonary fitness indicators than supine HRV measurements (Grant et al., 2009). For future studies it is recommended to investigate participants from other age groups as well as clinical groups to answer the question whether these results are only applicable to healthy young participants and if not; how it differs in other groups.

### Conflict of interest statement

The authors declare that the research was conducted in the absence of any commercial or financial relationships that could be construed as a potential conflict of interest.
